# Microbial diversity and potential functional dynamics within the rhizocompartments of *Dendrobium huoshanense*

**DOI:** 10.3389/fpls.2024.1450716

**Published:** 2024-09-20

**Authors:** Guijuan Xie, Zhichao Yin, Zhenlin Zhang, Xinyu Wang, Chuanbo Sun

**Affiliations:** ^1^ College of Biology and Pharmaceutical Engineering, West Anhui University, Lu’an, China; ^2^ Anhui Province Key Laboratory for Quality Evaluation and Improvement of Traditional Chinese Medicine, Lu’an, China; ^3^ Anhui Engineering Technology Center for Conservation and Utilization of Traditional Chinese Medicine Resource, Lu’an, China

**Keywords:** *Dendrobium huoshanense* microbiome, rhizocompartments, microbial diversity, niche width, high-throughput sequencing, endophytic fungi

## Abstract

**Introduction:**

Understanding the microbial diversity and potential functional dynamics within the rhizocompartments of *Dendrobium huoshanense* is crucial for unraveling the plant–microbe interactions that influence its medicinal properties.

**Methods:**

This study is the first to characterize the microbiome associated with the rhizocompartments of *D. huoshanense*, including its cultivation medium, rhizosphere, rhizoplane, and root endosphere, using high-throughput sequencing and subsequent bioinformatic analysis.

**Results:**

Bacterial phylogenetic diversity was significantly higher in the endosphere than in the rhizosphere, while fungal α-diversity significantly decreased from the cultivation medium to the endosphere. Both bacterial and fungal niche widths decreased from the cultivation medium to the endosphere. β-Diversity analysis revealed distinct spatial patterns in both bacterial and fungal communities across the rhizocompartments, with the most pronounced differences between the cultivation medium and the endosphere. Taxonomically, Proteobacteria and Ascomycota were predominant in the endosphere for bacterial and fungal communities, respectively. Functional predictions showed significant enrichment of pathways related to xenobiotics biodegradation, lipid metabolism, and nitrogen fixation in the endosphere, while functions associated with plant pathogens and saprotrophs were significantly reduced.

**Discussion:**

The results indicate a shift from generalist to specialist microbes from the cultivation medium to the endosphere, suggesting that *D. huoshanense* exerts strong selective pressure for endophytic fungi. Interestingly, a high proportion of fungi with unknown functions were found in the endosphere, highlighting an area for further research regarding the medicinal efficacy of *D. huoshanense*. Overall, this study provides foundational data for understanding the adaptive evolution of these microbial communities in response to specific microhabitats.

## 1 Introduction

*Dendrobium*, a genus within the Orchidaceae family, includes plants that are widely utilized in Chinese traditional medicine for their broad medicinal properties. These benefits encompass gastrointestinal nourishment, yin nourishment, heat clearance, lung moisture enhancement, cough relief, vision improvement, and body fortification (Shun et al., 2017; Gao et al., 2022). Consequently, *Dendrobium* is frequently consumed fresh as a dietary supplement or incorporated into traditional Chinese medicinal preparations. Modern pharmacological research has further unveiled additional benefits, including anti-aging, immune enhancement, anti-tumor effects, anti-atherosclerotic effects, and alleviation of rheumatoid arthritis (Xie et al., 2019; Fan et al., 2020; Shang et al., 2021; Ye et al., 2023). Huoshan County in Anhui Province, China, is noted as the earliest recorded habitat for *Dendrobium* in ancient herbal texts (Shun et al., 2017). The species *Dendrobium huoshanense* C.Z.Tang et S.J.Cheng, identified by Tang and Cheng (1984), is endemic to China, and its inclusion in the Chinese Pharmacopoeia in 2020 underscores its importance. Recognized as one of the superior species within the *Dendrobium* genus, *D. huoshanense* is prominent among the “Nine Immortal Herbs of China” and is renowned for its rich array of bioactive compounds, including polysaccharides, flavonoids, alkaloids, amino acids, phenols, and terpenoids, which contribute to its pharmacological efficacy (Li et al., 2020; Liu et al., 2021a; Gao et al., 2022).

The growth, development, and defense against pests and diseases of medicinal plants are intricately linked to their habitats and the endophytic microorganisms, particularly those residing in the rhizosphere (Jain et al., 2020; Ling et al., 2022). These interactions between plants and their associated microbiota are crucial for plant growth, quality, and overall health (Morgan et al., 2005; Köberl et al., 2013; Xu et al., 2023). Consequently, the rhizospheric microbiota are often referred to as a plant’s second genome. In addition to rhizospheric microbiota, endophytic fungi are essential symbiotic partners for orchids, significantly affecting the host plants through their life activities and secondary metabolites (Deng and Cao, 2017). Studies have suggested that the microbial community associated with *Dendrobium*, including both rhizospheric and endophytic components, plays a pivotal role in determining its quality. For example, *Sphingomonas paucimobilis* ZJSH1 has been shown to significantly promote the growth of *Dendrobium catenatum* and the accumulation of active polysaccharides (Yang et al., 2014; Li et al., 2023). Additionally, endophytic fungi isolated from *Dendrobium* exhibit antimicrobial and anti-inflammatory activities (Yue and Yu, 2022), with some fungi having the potential to enhance the growth of *D. huoshanense* or act as antagonists to plant pathogens (Chen et al., 2019).

Traditional microbial culture methods are limited by the inability to isolate and purify more than 95% of microorganisms (Amann et al., 1995; Rappé and Giovannoni, 2003), resulting in incomplete and often biased microbial profiles. In recent years, high-throughput sequencing technologies for prokaryotic 16S rRNA genes and fungal internal transcribed spacer (ITS) genes have emerged as critical tools for studying plant microbiomes. For instance, high-throughput sequencing has revealed higher diversity in root-associated bacteria and fungi in *D. huoshanense* compared to stems, with notable differences in bacterial diversity across different growth years (Chen et al., 2020). Liu et al. (2021b) employed high-throughput sequencing to investigate the diversity and composition of mycorrhizal fungi associated with three types of 1-year-old *D. huoshanense*, uncovering rich fungal diversity and significant differences among the types of *D. huoshanense*.

**Figure 1:**
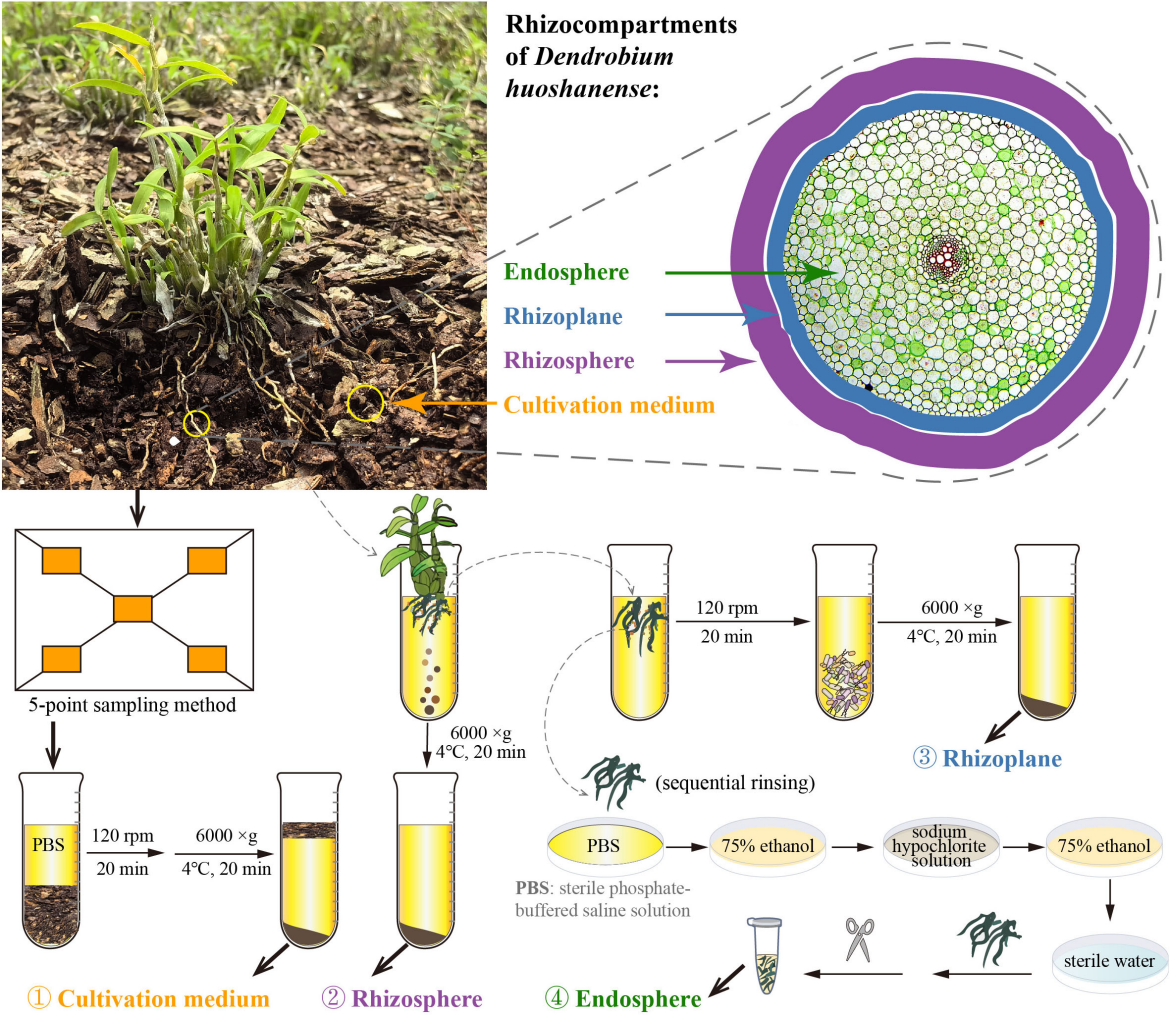
Sampling strategy diagram. Samples were taken at four compartments (different spatial categories), i.e., cultivation medium, rhizosphere, rhizoplane, and endosphere.

Plants are known to recruit specific groups of microorganisms to their rhizosphere, with only a subset penetrating the roots via the rhizoplane. These spatially heterogeneous microbial communities are shaped by complex plant–microbe interactions (Bulgarelli et al., 2013; Edwards et al., 2015; Attia et al., 2022). However, very little is known about the differences in the rhizosphere, rhizoplane, and root endophytic microbiomes of *D. huoshanense* and their potential functions, which severely limits our understanding of the interaction mechanisms between *D. huoshanense* and microorganisms. To address these research gaps, this study systematically investigated the root-associated microbiome of *D. huoshanense* across four distinct rhizocompartments—the cultivation medium, rhizosphere, rhizoplane, and endosphere (**Figure 1**)—using high-throughput sequencing technology. The primary objectives were to elucidate the characteristics of bacterial and fungal diversity, observe community structure changes across these rhizocompartments, and identify potential functional differences among the bacterial and fungal communities. These findings will contribute to a deeper understanding of interactions between medicinal plants, rhizospheric microorganisms, and endophytic microorganisms, thereby enriching the theoretical foundation for traditional medicinal plant–microbe interactions.

## 2 Materials and methods

### 2.1 Experimental setup and sample collection

Wild *D. huoshanense* is a rare and authentic medicinal herb found only in certain regions of the Dabie Mountains. Historically, it was threatened with extinction due to overharvesting. Since the beginning of this century, with strong support from local governments and the concerted efforts of research teams from West Anhui University and numerous enterprises, the artificially cultivated resources of *D. huoshanense* have been restored. Currently, there is a 6,000-m^2^ cultivation base for *D. huoshanense* at West Anhui University, with the planting medium consisting of pine bark, pine dust, and a small amount of sandstone gravel. As a perennial herb, *D. huoshanense* is typically harvested every 3 years for medicinal use (Chen et al., 2020). Research has shown that the polysaccharide, alkaloid, and amino acid contents of 3-year-old *D. huoshanense* are higher compared to those of plants at other ages (Yuan et al., 2019).

In this study, we selected healthy 3-year-old *D. huoshanense* as the research subject and conducted sample collection on March 22, 2023, at the *D. huoshanense* cultivation base in the Medicinal Plant Garden of West Anhui University. The specific sampling steps were as follows (**Figure 1**). 1) Collection of cultivation medium samples: the five-point sampling method was employed (He et al., 2021). Using a sterilized shovel, *D. huoshanense* growth medium was collected and divided into six sterile kraft paper bags as biological replicates. The physical and chemical parameters of the cultivation medium are listed in **Supplementary Table S1**. 2) Collection of rhizosphere and rhizoplane samples: six clusters of *D. huoshanense* were gently lifted with a sterile shovel, and loose growth mediums were shaken off, leaving the mediums near the roots. Rhizosphere and rhizoplane samples were collected using sterile sampling bags and transported to the laboratory. Soil adhering to the roots, approximately 1 mm thick, was defined as rhizospheric soil (Edwards et al., 2015). Bulk mediums adhering to the roots were shaken off within a laminar flow hood, and mediums still adhering to the roots were considered rhizosphere. The roots were rinsed with sterile phosphate-buffered saline (PBS) solution, and the rinse solution was collected. After centrifugation at 6,000 *g* for 20 minutes at 4°C, the collected rhizospheric samples were obtained. Then, approximately 4.5 g of root samples was transferred to sterile 50-mL centrifuge tubes containing 20 mL of sterile 10 mM PBS solution and shaken at 120 rpm at room temperature for 20 minutes (Beckers et al., 2016a, 2017). Using sterile forceps, roots were removed from the tubes, and the remaining suspension was centrifuged (6,000 *g*, 4°C) for 20 minutes to collect rhizoplane samples. 3) Collection of endosphere samples: approximately 4.5 g of *Dendrobium* roots previously rinsed with PBS solution, dried, washed with sterile water, surface moisture absorbed, soaked in 75% (v/v) ethanol for 2 minutes, washed with sodium hypochlorite solution for 5 minutes, soaked again in 75% ethanol for 30 seconds, and rinsed with sterile water five times was transferred into sterile 2-mL centrifuge tubes using sterile scissors in a laminar flow hood (Baker, Sanford, ME, USA) to serve as endosphere samples (Beckers et al., 2017). Six biological replicates of each cultivation medium, rhizosphere, rhizoplane, and endosphere samples were collected and stored at −80°C for DNA extraction.

### 2.2 DNA extraction and Illumina MiSeq sequencing

DNA was extracted using the FastDNA^®^ Spin Kit for Soil (MP Biomedicals, Santa Ana, CA, USA) following a bead-beating procedure (homogenization for 20 minutes at 3,000 rpm) (Cullen et al., 2022). The hypervariable V5–V7 region of the bacterial 16S rRNA gene was amplified using the primer set 799F (5′-AACMGGATTAGATACCCKG-3′) and 1193R (5′-ACGTCATCCCCACCTTCC-3′) (Beckers et al., 2016b). Fungal internal transcribed spacer region 2 (ITS2) was amplified using the primer set ITS3F (5′-GCATCGATGAAGAACGCAGC-3′) and ITS4R (5′-TCCTCCGCTTATTGATATGC-3′) (White et al., 1990). Paired-end sequencing (2 × 250 bp) was performed on the Illumina MiSeq platform at Guangdong Magigene Biotechnology (Guangzhou, China).

### 2.3 Bioinformatics processing

Sequencing analysis was conducted using QIIME 2 version 2024.2 (Bolyen et al., 2019). Raw sequencing reads were demultiplexed and quality filtered with the q2‐demux plugin, followed by denoising using DADA2 (Callahan et al., 2016). Chimeric sequences were removed from subsequent analyses. Amplicon sequence variants (ASVs) with fewer than 10 reads were excluded to mitigate sequencing errors. Representative ASVs were aligned with MAFFT (Katoh et al., 2002) and used to construct a phylogeny via FastTree2 (Price et al., 2010). Taxonomic assignments were conducted using the classify‐sklearn naiüve Bayes taxonomy classifier (Bokulich et al., 2018) against the SILVA database (release 138) for bacteria (Quast et al., 2013) and UNITE database v10.0 for fungi (Abarenkov et al., 2024). Microbial diversity indices, including observed ASVs (ASV richness), Faith’s phylogenetic diversity (Faith, 1992), and the Shannon index, were calculated using uniform sequencing depths of 46,832 for bacteria and 88,813 for fungi. Rarefaction curves were constructed to assess α-diversity and estimate sequencing depth.

### 2.4 Taxonomic composition and putative functional analysis

Taxonomy profiles from the phylum to genus level, based on ASVs with >0.1% relative abundance for bacteria and >0.05% for fungi, were selected for differential analysis across the four rhizocompartments. The identification of significantly different distributions of bacterial and fungal taxa among these rhizocompartments was performed using linear discriminant analysis effect size (LEfSe), with a threshold of 4.0 for linear discriminant analysis (LDA) scores and a significance level of 0.01 (Segata et al., 2011).

For bacteria, putative functional categories were annotated using Phylogenetic Investigation of Communities by Reconstruction of Unobserved States (PICRUSt2) v2.5.2 (Douglas et al., 2020) and the Functional Annotation of Prokaryotic Taxa (FAPROTAX) v1.2.6 database (Louca et al., 2016). Fungal function prediction was conducted using the FungalTraits database (Põlme et al., 2020), where each fungal genus was assigned a functional guild based on its “primary lifestyle”. Statistical analysis for multiple comparisons of the relative abundance of predicted functional categories/guilds was carried out using STAMP v2.1.3 (Parks et al., 2014), employing non-parametric Kruskal–Wallis tests with the Holm–Bonferroni correction for *post-hoc* tests (Benjamini and Hochberg, 1995).

Both LEfSe and FAPROTAX analyses were performed using the online platform available at https://www.bic.ac.cn/ImageGP/ (Chen et al., 2022). The significant functional categories/guilds among rhizocompartments were visualized using the “pheatmap” package v1.0.12 in R version 4.3.2, with scaled relative abundances.

### 2.5 Statistical analysis

To assess differences in α-diversity indices across the four rhizocompartments, non-parametric Kruskal–Wallis tests were conducted using the “*boxplerk()*” function developed by Borcard et al. (2018), with the Holm–Bonferroni correction applied for multiple comparisons in R version 4.3.2. To elucidate the spatial distribution patterns of microbial communities within *D. huoshanense* microhabitats, ASV tables were log(*x* + 1) transformed, followed by metric multidimensional scaling (mMDS) based on the Bray–Curtis similarity using PRIMER v7 (Clarke and Gorley, 2015). Non-parametric analysis of similarity (ANOSIM) was employed to evaluate the statistical differences among rhizocompartments, employing 999 permutations (Clarke and Green, 1988; Somerfield et al., 2021). Levins’ niche width (Levins, 1968) of bacterial and fungal communities across different rhizocompartments was computed using the “spaa” package v0.2.2.

## 3 Results

### 3.1 Microbial α-diversity across spatially distinct rhizocompartments

**Figure 2:**
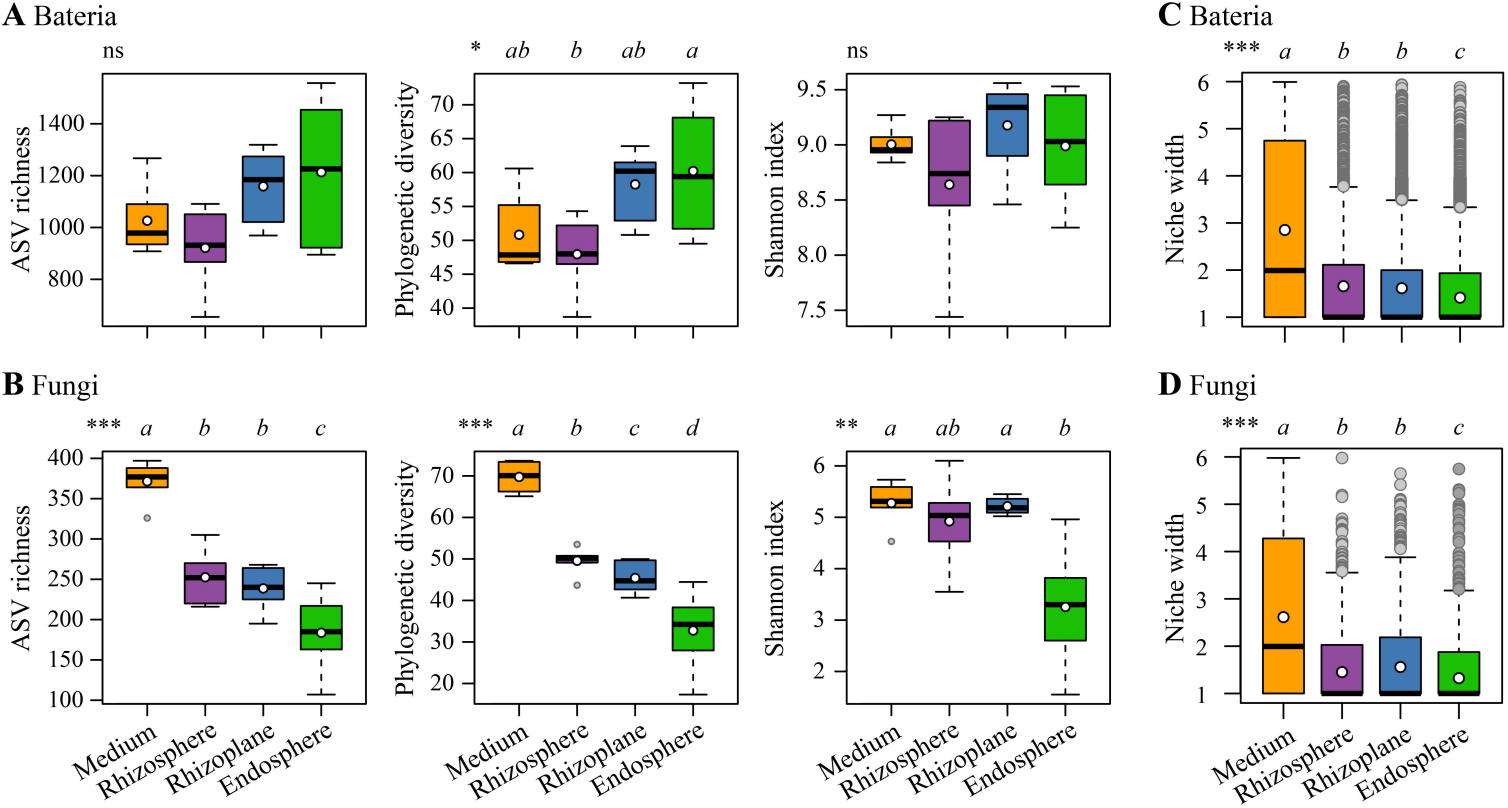
Comparisons of α-diversity indices for bacterial communities **(A)** and fungal communities **(B)**, as well as niche width for bacterial communities **(C)** and fungal communities **(D)** across different compartments (the cultivation medium, rhizosphere, rhizoplane, and endosphere). Different lowercase letters above each boxplot indicate significant differences, determined by the Kruskal–Wallis test with Holm corrections. Overall significance is denoted as follows: ns, non-significant; *p < 0.05; **p < 0.01; ***p < 0.001. In the boxplot, the bold short black line and white dot denote the median and the mean of each index, respectively.

Rarefaction curves of both bacterial and fungal diversity data of the samples reached the saturation plateau, indicating sufficient sequencing depth (**Supplementary Figure 1**). For bacterial communities, the endosphere exhibited significantly higher phylogenetic diversity compared to the rhizosphere (*p* < 0.05, **Figure 2A**). While there was an increasing trend in ASV richness from the rhizosphere to the endosphere, the differences were not statistically significant. Similarly, differences in the Shannon index across the four rhizocompartments were not significant. In fungal communities, there was a distinct decreasing trend in ASV richness and phylogenetic diversity from the cultivation medium to the endosphere (*p* < 0.001, **Figure 2B**). Additionally, the Shannon index in the endosphere was significantly lower than that in the other three rhizocompartments. The niche width for both bacterial and fungal communities exhibited a decreasing trend from the cultivation medium to the endosphere of *D. huoshanense* (*p* < 0.001, **Figures 2C, D**).

### 3.2 Microbial β-diversity across spatially distinct rhizocompartments

**Figure 3:**
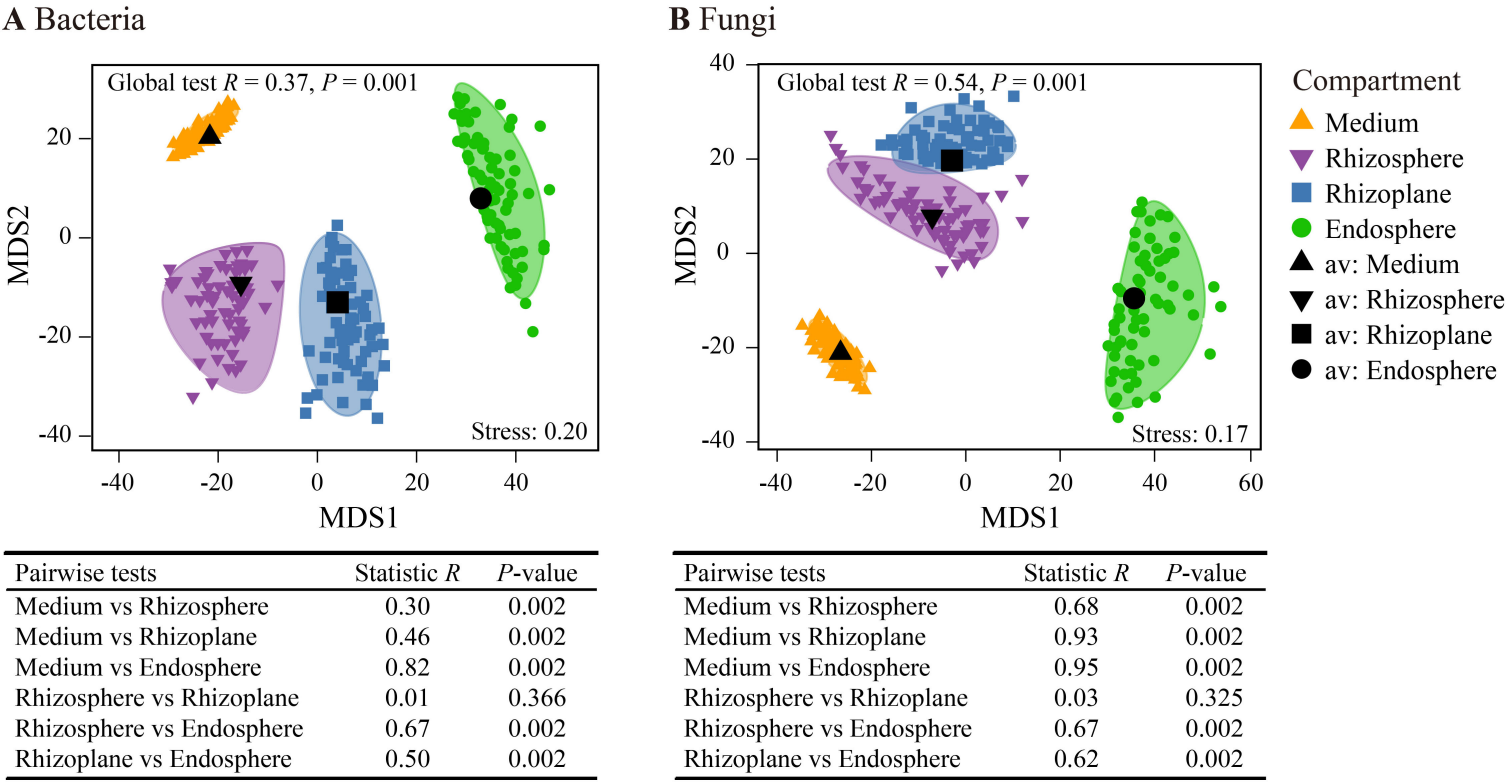
Metric multidimensional scaling plots (mMDS) of bootstrap averages (100 repetitions) for bacterial communities **(A)** and fungal communities **(B)** showing the spatial variation of community structures based on Bray–Curtis distance. Smooth envelopes indicate more than 80% confidence intervals for each compartment, with the centroid of each compartment marked in black. The dots inside and around the colored area represent the individual bootstrap values. Analysis of similarity (ANOSIM) was employed to test the significance of differences across the four compartments.

The mMDS bootstrap average analysis revealed distinct spatial patterns in both bacterial and fungal communities across the four rhizocompartments (**Figure 3**). ANOSIM indicated significant microbial variations across the spatially distinct rhizocompartments (*p* = 0.001). Pairwise tests showed significant differences in both bacterial and fungal communities between each pair of rhizocompartments, except for the rhizosphere and rhizoplane. Furthermore, the highest *R* statistic was observed between the cultivation medium and the endosphere in both bacterial and fungal communities, indicating the most pronounced differences between these two rhizocompartments.

### 3.3 Microbial taxonomic compositions across spatially distinct rhizocompartments

**Figure 4:**
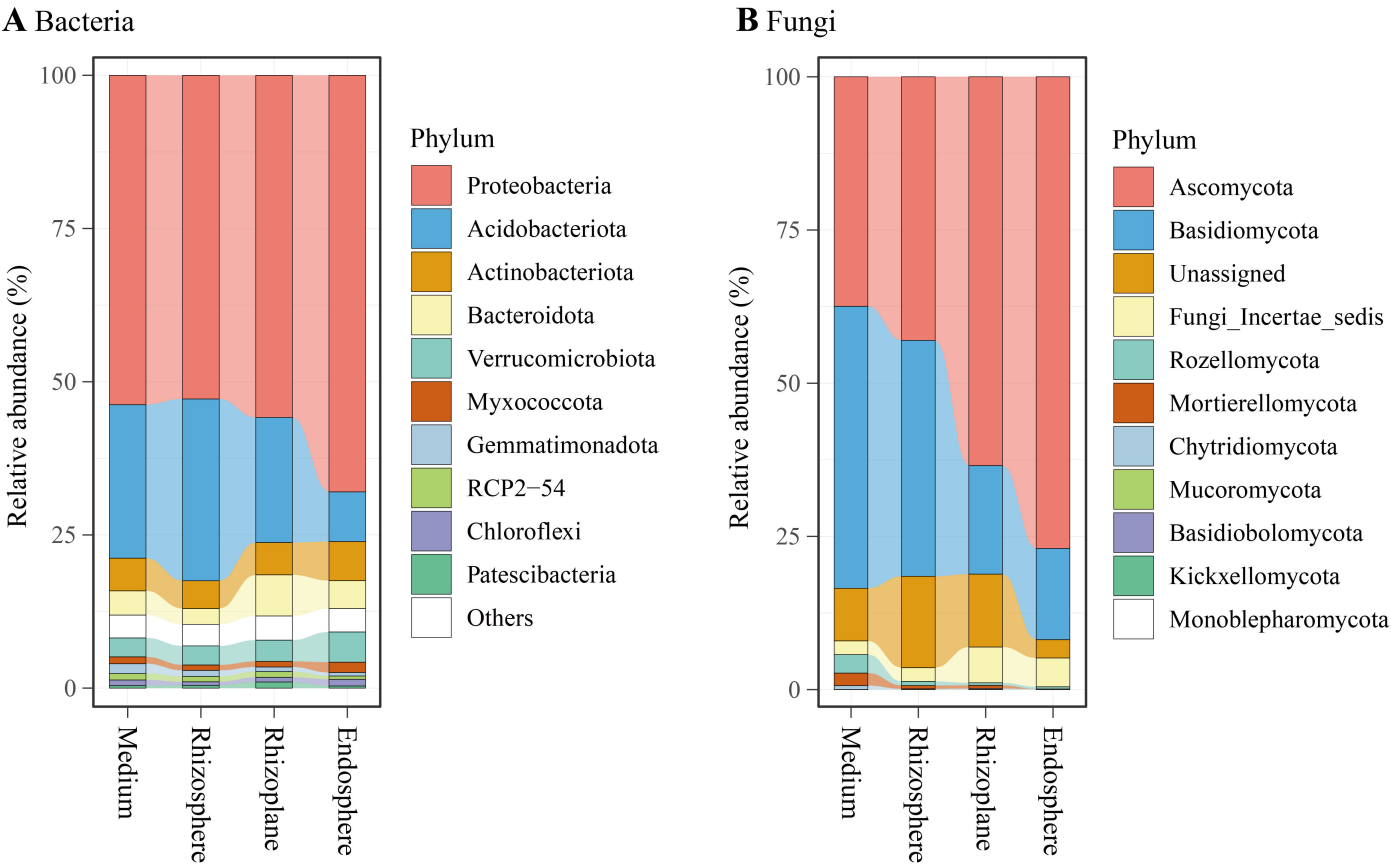
Relative abundances of major bacterial phyla **(A)** and fungal phyla **(B)** associated with the cultivation medium, rhizosphere, rhizoplane, and endosphere of *Dendrobium huoshanense*.

**Figure 5:**
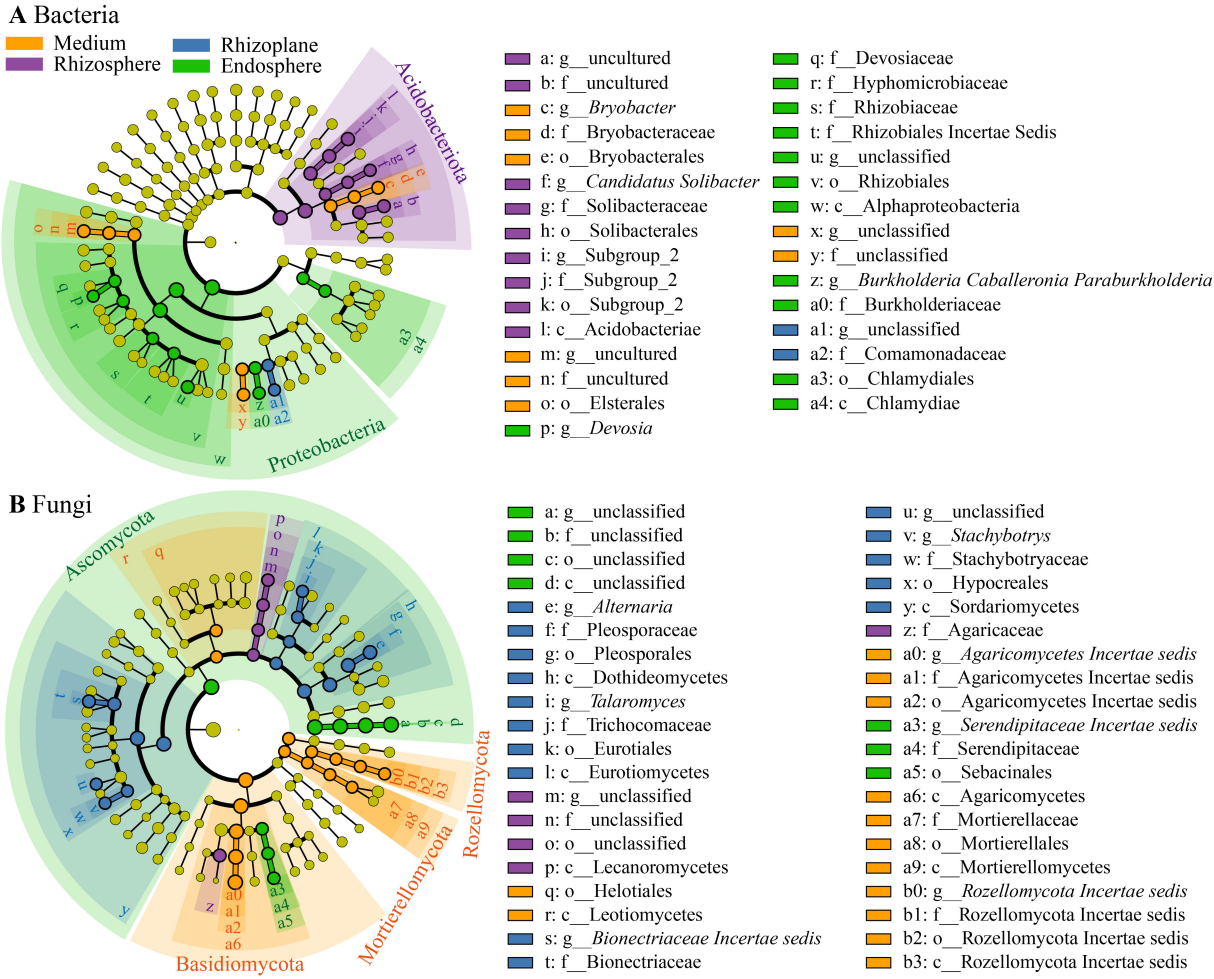
Cladogram generated through linear discriminant analysis effect size (LEfSe) analysis illustrating the differences in bacterial taxa **(A)** and fungal taxa **(B)**, from phyla (outermost of the ring) to genera (innermost), across the four compartments. Circles colored yellow, purple, blue, and green denote taxa significantly enriched in the cultivation medium, rhizosphere, rhizoplane, and endosphere, respectively.

Bacterial reads were classified into 35 phyla (including an unassigned phylum), 92 classes (c), 205 orders (o), 334 families (f), 595 genera (g), and 6,348 ASVs. At the phylum level (**Figure 4A**), Proteobacteria dominated the bacterial community (57.6%), followed by Acidobacteriota (20.8%), Actinobacteria (5.4%), Bacteroidota (4.5%), Verrucomicrobiota (3.7%), and Myxococcota (1.1%). LEfSe analysis revealed an enrichment of Proteobacteria in the endosphere (68.0% of total reads), while Acidobacteriota was enriched in the rhizosphere (**Figure 5A**). At the class level, Alphaproteobacteria (*w* in **Figure 5A**) and Chlamydiae (*a4*) were enriched in the endosphere, whereas Acidobacteriae (*l*) predominated in the rhizosphere. At the genus level (**Figure 5A**; **Supplementary Figure 2A**), the endosphere exhibited enrichment of *Devosia* (*p*), and *Burkholderia*, *Caballeronia*, and *Paraburkholderia* (*z*), while the cultivation medium favored *Bryobacter* (*c*), and the rhizosphere showed enrichment of *Candidatus Solibacter* (*f*). Additionally, the family Comamonadaceae (*a2*) was enriched in the rhizoplane.

Fungal reads were categorized into 11 phyla, 31 classes (c), 71 orders (o), 133 families (f), 205 genera (g), and 1,370 ASVs. At the phylum level (**Figure 4B**), Ascomycota dominated the fungal community (55.2%), followed by Basidiomycota (29.3%), Unassigned (9.6%), Fungi Incertae sedis (3.8%), and Rozellomycota (1.1%). LEfSe analysis indicated an enrichment of Ascomycota in the endosphere (77.0% of total reads), while Basidiomycota, Rozellomycota, and Mortierellomycota were enriched in the cultivation medium (**Figure 5B**). At the class level, Sebacinales (*a5*) and an unclassified class from Ascomycota (*d*) were enriched in the endosphere, while Leotiomycetes (*r*), Agaricomycetes (*a6*), Mortierellomycetes (*a9*), and Rozellomycota Incertae sedis (*b3*) were enriched in the cultivation medium. The rhizosphere exhibited enrichment of Lecanoromycetes (*p*), whereas the rhizoplane showed enrichment of Dothideomycetes (*h*), Eurotiomycetes (*l*), and Sordariomycetes (*y*). At the genus level (**Figure 5B**; **Supplementary Figure 2B**), the endosphere was enriched with *Serendipitaceae Incertae sedis* (*a3*), while the rhizoplane showed enrichment of *Alternaria* (*e*), *Talaromyces* (*i*), *Bionectriaceae Incertae sedis* (*s*), and *Stachybotrys* (*v*); the cultivation medium favored *Agaricomycetes Incertae sedis* (*a0*) and *Rozellomycota Incertae sedis* (*b0*). Within Ascomycota, the genus *Fusarium* exhibited distinct higher relative abundance in the rhizoplane and endosphere compared to the medium and rhizosphere (**Supplementary Figure 2B**). Additionally, our study revealed that the rhizocompartments of *D. huoshanense*, particularly within its roots, harbor a substantial number of microorganisms that are not annotated to the genus or species level.

### 3.4 Putative functional compositions across spatially distinct rhizocompartments

**Figure 6:**
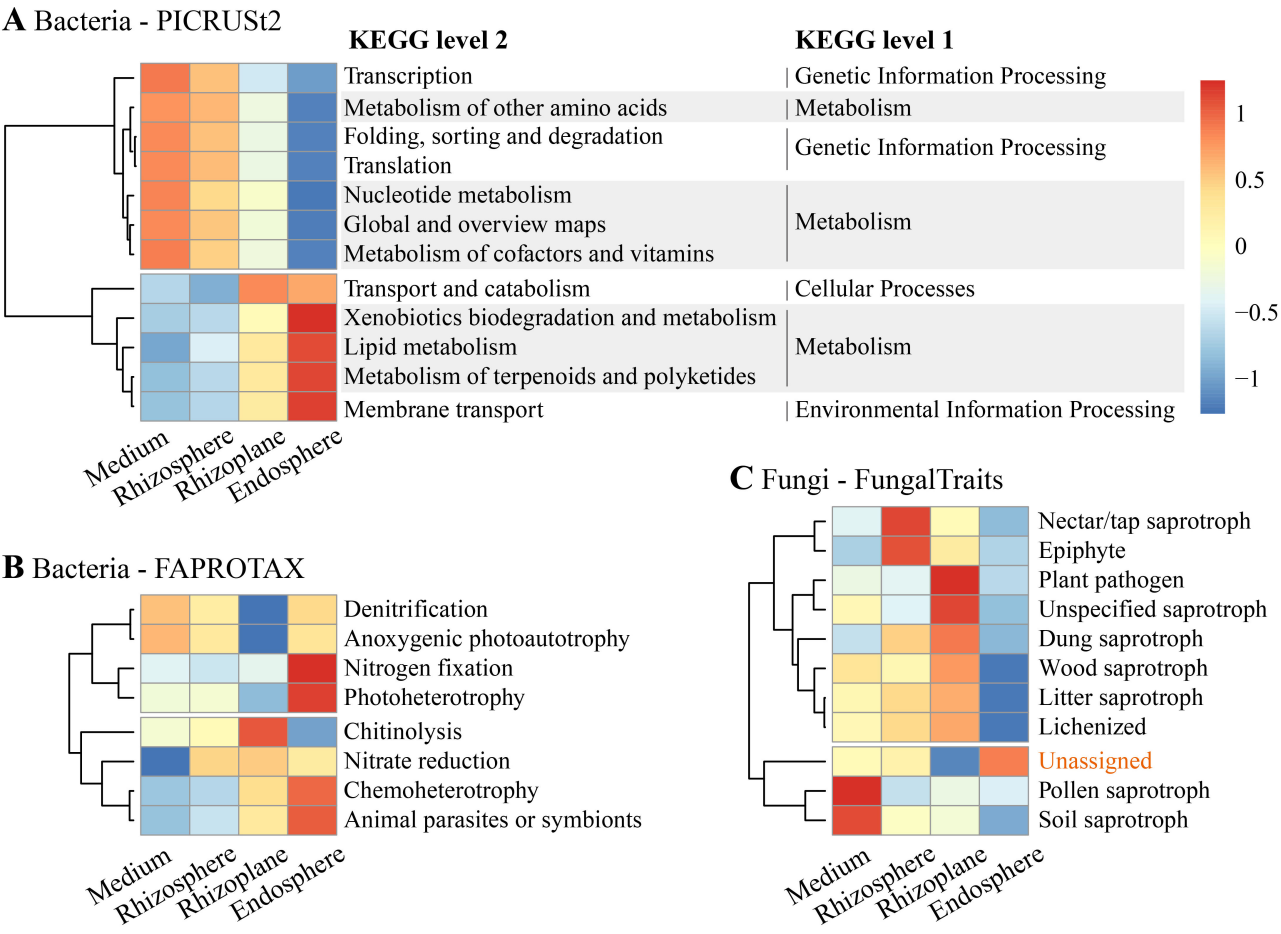
Functional profiles showing significant differences in functional categories/guilds for bacterial communities annotated using **(A)** Phylogenetic Investigation of Communities by Reconstruction of Unobserved States (PICRUSt2) and **(B)** Functional Annotation of Prokaryotic Taxa (FAPROTAX), as well as **(C)** for fungal communities using FungalTraits, across the four compartments.

Using PICRUSt2, we identified significant differences in 12 predicted bacterial Kyoto Encyclopedia of Genes and Genomes (KEGG) level 2 pathways across the four rhizocompartments (all *p* < 0.01, **Figure 6A**). Among these pathways, xenobiotics biodegradation and metabolism, lipid metabolism, metabolism of terpenoids and polyketides, and membrane transport were enriched in the endosphere. Conversely, transcription, metabolism of other amino acids, folding, sorting and degradation, translation, nucleotide metabolism, global and overview maps, and metabolism of cofactors and vitamins were enriched in the cultivation medium. FAPROTAX analysis revealed significant enrichment of putative functions related to nitrogen fixation, photoheterotrophy, chemoheterotrophy, and animal parasites or symbionts in the endosphere (all *p* < 0.05, **Figure 6B**). Chitinolysis was notably enriched in the rhizoplane, while denitrification and anoxygenic photoautotrophy were depleted in the rhizoplane.

Regarding fungal communities, functional guilds associated with pollen saprotroph and soil saprotroph were significantly enriched in the cultivation medium (*p* < 0.01), while nectar/tap saprotroph and epiphyte were enriched in the rhizosphere (*p* < 0.05) (**Figure 6C**). Plant pathogens, unspecified saprotrophs, dung saprotrophs, wood saprotrophs, litter saprotrophs, and lichenized were significantly enriched in the rhizoplane. Notably, functional guilds associated with all types of saprotrophs were depleted in the endosphere. Additionally, reads with unassigned functions were significantly enriched in the endosphere, constituting 87% of the total reads in this compartment.

## 4 Discussion

### 4.1 Bacterial community diversity and composition across rhizocompartments of *D. huoshanense*

Our results demonstrated an increasing phylogenetic diversity of bacteria from the rhizosphere to the root endosphere of *D. huoshanense* (**Figure 2**). The bacterial α-diversity pattern across rhizocompartments of *D. huoshanense* differs from that observed in other plants. For instance, the characterization of the root microbiome of *Arabidopsis* revealed that bacterial diversity inside the root (the endosphere) was much lower compared to the soil around the root (the rhizosphere) (Schlaeppi et al., 2014). Similarly, bacterial richness in the rhizoplane and rhizosphere of wheat and faba bean was significantly lower than that in the soil (Attia et al., 2022). The richness of bacteria in the endosphere of roots, stems, and leaves of poplar trees was also significantly lower than that in rhizosphere soil (Beckers et al., 2017). These variations in root-associated bacterial diversity patterns may be related to the plant’s growth environment. The artificial cultivation medium of *D. huoshanense* in this study differs from natural soil, potentially resulting in lower bacterial diversity in the cultivation medium due to lower concentrations of nutrients. In contrast, the relatively abundant nutrients at the root surface and interior may lead to increased bacterial diversity. Additionally, the niche width of bacterial communities within the roots of *D. huoshanense* was significantly narrower than that in the growth medium, rhizosphere, and rhizoplane (**Figure 2C**). This indicates a gradual shift from generalists to specialists as bacteria transition from the medium to the root interior, adapting to the increasingly specific microenvironment (Lambers et al., 2009; Beckers et al., 2017).

Regarding bacterial community composition, the relative abundance of Proteobacteria was significantly enriched in the endophytic environment of *D. huoshanense* roots (**Figure 5A**). This finding is consistent with the results of Chen et al. (2020). At the genus level, *Devosia* was also significantly enriched in the endosphere of *D. huoshanense* (**Figure 5A**, **Supplementary Figure 2A**). *Devosia* consists of rod-shaped, motile, gram-negative soil bacteria capable of symbiotically fixing atmospheric nitrogen (Hungria and Nogueira, 2023), decomposing urea, and producing various volatile organic compounds, which contribute to soil microflora diversity and mediate microbe–microbe interactions (Schenkel et al., 2015). Genomic analysis further revealed *Devosia*’s ability to sense environmental signals and exhibit chemotaxis in stressed habitats (Talwar et al., 2020). The enrichment of *Devosia* in the endosphere of *D. huoshanense* may positively affect the plant’s growth and adaptation to stressed environments. Another notable feature in the community composition is that the relative abundance of Acidobacteriota within the roots of *D. huoshanense* is much lower than in other rhizocompartments, especially in the rhizosphere (**Figure 4**). Many Acidobacteriota are acidophilic and abundant in soil habitats (Kielak et al., 2016). The high abundance of Acidobacteriota in the cultivation medium, rhizosphere, and rhizoplane observed in this study may be related to the low pH value of the growth substrate for *D. huoshanense* (**Supplementary Table S1**).

### 4.2 Fungal community diversity and composition across rhizocompartments of *D. huoshanense*

The richness and phylogenetic diversity of fungi within the roots of *D. huoshanense* are significantly lower than those in other rhizocompartments (**Figure 2B**). Studies have shown that fungal diversity decreases progressively from the bulk soil to the root interior (Mendes et al., 2013; Hardoim et al., 2015; Bahram et al., 2018). This gradient is shaped by nutrient availability, environmental conditions, and the selective pressure exerted by plant roots. The bulk soil supports a rich and diverse fungal community due to its complexity and variety of niches (Tedersoo et al., 2014). In contrast, the rhizosphere and rhizoplane select fungi that can utilize root exudates and adhere to root surfaces, respectively (Berendsen et al., 2012; Li et al., 2021). Finally, the endosphere has the lowest fungal diversity because it is inhabited only by specific fungi capable of overcoming plant defenses and establishing symbiotic or endophytic relationships with their hosts (Hardoim et al., 2015; Qian et al., 2019; Sonam and Liu, 2024). Additionally, the niche width of fungal communities within the roots of *D. huoshanense* was significantly narrower than that in the growth medium, rhizosphere, and rhizoplane (**Figure 2D**). This also reflects the adaptation of specialist fungi to the increasingly specialized microenvironment (Huang et al., 2022; Sonam and Liu, 2024).

Regarding fungal community composition, the most significant feature is the substantial increase in the relative abundance of Ascomycota from the cultivation medium to the interior of *D. huoshanense* roots, while the relative abundance of Basidiomycota significantly decreases (**Figure 4B**). The enrichment of Ascomycota within the endosphere of *D. huoshanense* is mainly due to the high abundance of an unclassified genus (labeled “a” in **Figure 5B**; **Supplementary Figure 2B**). This fungal genus accounts for up to 62.2% of the endosphere, whereas its proportion in the other three rhizocompartments does not exceed 6.0%. Due to the lack of taxonomic information and culturable strains, the function and ecological role of this fungal taxon remain to be further studied. Although fungal community composition varies across different plant-associated environments, bulk soil and the rhizosphere are dominated by Ascomycota, Basidiomycota, and Zygomycota (Berendsen et al., 2012; Tedersoo et al., 2014; Bahram et al., 2018). In contrast, the endosphere is primarily dominated by Ascomycota, with fewer Basidiomycota (Hardoim et al., 2015). *Fusarium* spp. were enriched in the rhizoplane and endosphere (**Supplementary Figure 2B**). They are filamentous fungi commonly found in soil and plant debris (Fravel et al., 2003). Most of them are harmless saprobes, although some species can be pathogenic and produce mycotoxins (Ma et al., 2010). Ecologically, they play a role in decomposing organic matter and nutrient cycling (Chen et al., 2014). Some species have beneficial interactions, such as promoting plant growth or acting as biocontrol agents against other pathogens (Nikitin et al., 2023).

### 4.3 Bacterial and fungal community structures across rhizocompartments of *D. huoshanense*

Bacterial and fungal community structures (β-diversity) of *D. huoshanense* in the rhizosphere and rhizoplane showed no significant differences (**Figure 3**). This may be attributed to the cultivation medium of *D. huoshanense*, which mainly consists of pine bark. This environment differs from soil environments, resulting in smaller differences in the microenvironments of the rhizosphere and rhizoplane of *D. huoshanense*, leading to minimal spatial differentiation. However, the bacterial and fungal community structures of the rhizosphere and rhizoplane of *D. huoshanense* differed significantly from those in the cultivation medium and the internal root environment of *D. huoshanense*, indicating that different microhabitats have a substantial impact on microbial community structure. This finding is consistent with the results reported by Sonam and Liu (2024). Additionally, there were considerable differences in the endophytic community structures within the roots of different *D. huoshanense* plants (**Figure 3**), suggesting that environmental selection by individual plants strongly determines local microbial communities of the plant endosphere (Attia et al., 2022).

### 4.4 Microbial functional differences across rhizocompartments of *D. huoshanense*

Bacterial functional analysis indicates that metabolic processes related to xenobiotics, lipids, and functions associated with nutrient acquisition (e.g., nitrogen fixation) are significantly enriched in the *D. huoshanense* endosphere (**Figure 6**). The enrichment of these functions may be causally related to the significant enrichment of membrane transport, symbionts, and chemoheterotrophy (**Figure 6**). This suggests that *D. huoshanense* may selectively enrich bacterial groups with these functions through interactions with environmental bacteria, thereby promoting its own growth, enhancing specific metabolic pathways, and facilitating the formation of bioactive macromolecules (Yang et al., 2014; Yu and Hochholdinger, 2018; Li et al., 2023; Ravelo-Ortega et al., 2023). For example, the enrichment of terpenoid alkaloid metabolic functions in the endosphere of *D. huoshanense* (**Figure 6**) may related to the high content of medicinally active alkaloid metabolites in *D. huoshanense* (Wu et al., 2022).

FungalTraits analysis indicated that functions associated with plant pathogens and saprotrophs were significantly depleted in the endosphere, while these functions were abundant in the growth medium, rhizosphere, or rhizoplane (**Figure 6C**). These results suggest that the roots of *D. huoshanense* serve as a crucial barrier in interactions with environmental fungi (Li et al., 2021). This barrier restricts the entry of pathogens into the roots and prevents hyper-decomposing saprophytic fungi from bypassing the root barrier, effectively protecting *D. huoshanense* from both pathogenic and saprophytic fungi. For instance, the relative abundance of *Alternaria* in the external root environment (from the growth medium to the rhizoplane) ranges from 6.3% to 15.3%, whereas in the endosphere, it is only approximately 0.1% (**Supplementary Figure 2**). *Alternaria*, a genus of Deuteromycetes fungi, includes species that are major plant pathogens and common agents of decay and decomposition, as well as producers of mycotoxin (Thomma, 2003; Du et al., 2023).

In this study, up to 87% of fungal sequences within the roots remain functionally uncharacterized. This greatly limits our understanding of the structure and function of the *D. huoshanense* holobiome (Hardoim et al., 2015). Future research should employ advanced methods such as metagenomics, metabolomics, and culturomics (Rinke et al., 2013; Mhlongo et al., 2018; Matar and Bilen, 2022) to further investigate the ecological functions of dominant microbial groups in the rhizosphere and within the roots.

## 5 Conclusions

In summary, our study represents the first comprehensive characterization of the microbiome associated with the rhizocompartments of *D. huoshanense* using high-throughput sequencing. Our findings revealed several key insights: first, fungal α-diversity exhibits a significant decrease from the cultivation medium to the root endosphere, while bacterial α-diversity peaks in the endosphere. Moreover, both bacterial and fungal community niche widths contract from the cultivation medium to the endosphere, indicating a shift toward more specialized microbial communities. Second, the distinct structures and taxonomic compositions observed in the bacterial and fungal communities within the roots, compared to other rhizocompartments, highlight adaptive evolution toward specific microhabitats. Lastly, we observed a notable reduction in functions associated with plant pathogens and saprotrophs within the root microbiome, indicating strong selective pressures favoring endophytic fungi beneficial to *D. huoshanense*. Conversely, the endosphere is enriched with fungi of unknown function, underscoring the need for further investigation into their potential roles in influencing the medicinal properties of *D. huoshanense*. Overall, our study not only provides novel insights into the microbial ecology of *D. huoshanense* but also underscores the complexity and specificity of its endophytic microbiome.

## Data Availability

The datasets presented in this study can be found in online repositories. The names of the repository/repositories and accession number(s) can be found in the article/**Supplementary Material**.
