# Supplementary material for: Microbial diversity and potential functional dynamics within the rhizocompartments of Dendrobium huoshanense
Source: Front Plant Sci. 2024 Sep 20;15:1450716. doi: 10.3389/fpls.2024.1450716 (PMC11449778; doi:10.3389/fpls.2024.1450716)
Supplement: Supplementary file 1 [file DataSheet1.docx]

**Supplementary Materials for:**

**Microbial diversity and** **potential functional dynamics within the rhizocompartments of *Dendrobium huoshanense***

Guijuan Xie^1, 2, 3^, Zhichao Yin^1^, Zhenlin Zhang^1^, Xinyu Wang^1^, Chuanbo Sun^1, 2, 3 *^

^1^ College of Biology and Pharmaceutical Engineering, West Anhui University, Lu’an 237012, China;

^2^ Anhui Province Key Laboratory for Quality Evaluation and Improvement of Traditional Chinese Medicine, Lu’an City 237012, China;

^3^ Anhui Engineering Technology Center for Conservation and Utilization of Traditional Chinese Medicine Resource, Lu’an City 237012, China.

*** Corresponding author:**

Chuanbo Sun

[scb19781979@126.com](mailto:scb19781979@126.com)

**Supplementary Table S1** Physical and chemical parameters of the cultivation medium for *Dendrobium huoshanense*

| Parameters | Mean | Standard deviation | Range |
| --- | --- | --- | --- |
| pH | 5.83 | 0.41 | 5.41~6.42 |
| Moisture content (%) | 34.1 | 2.9 | 29.9~38.3 |
| Organic matter content (%) | 59.6 | 10.6 | 39.9~70.1 |


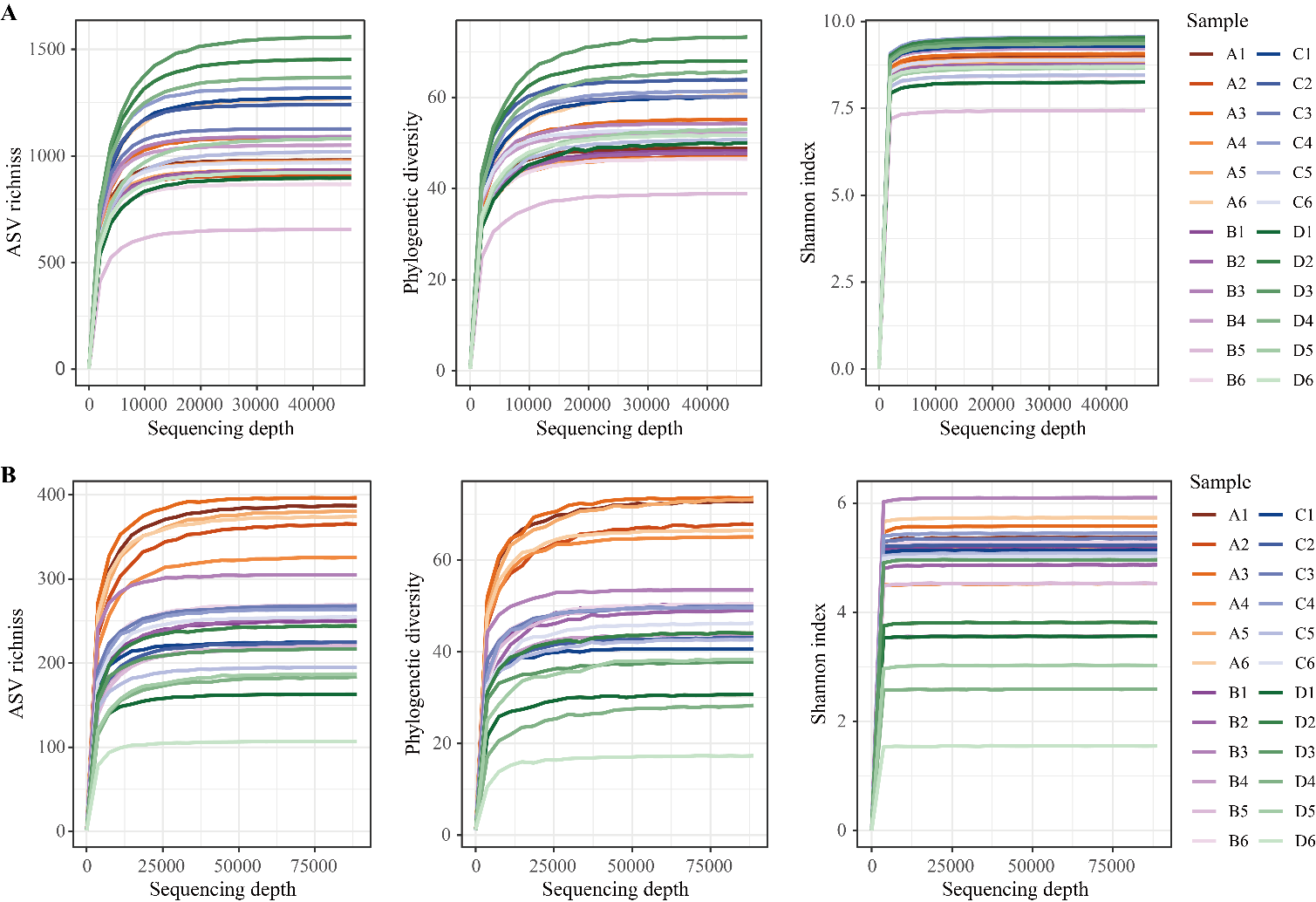


**Supplementary Figure 1** Rarefaction curves of alpha-diversity indices showing the sequencing depth of bacterial (**A**) and fungal (**B**) data obtained from the cultivation medium (A1-A6), rhizosphere (B1-B6), rhizoplane (C1-C6), and endosphere (D1-D6) of *D. huoshanense*.
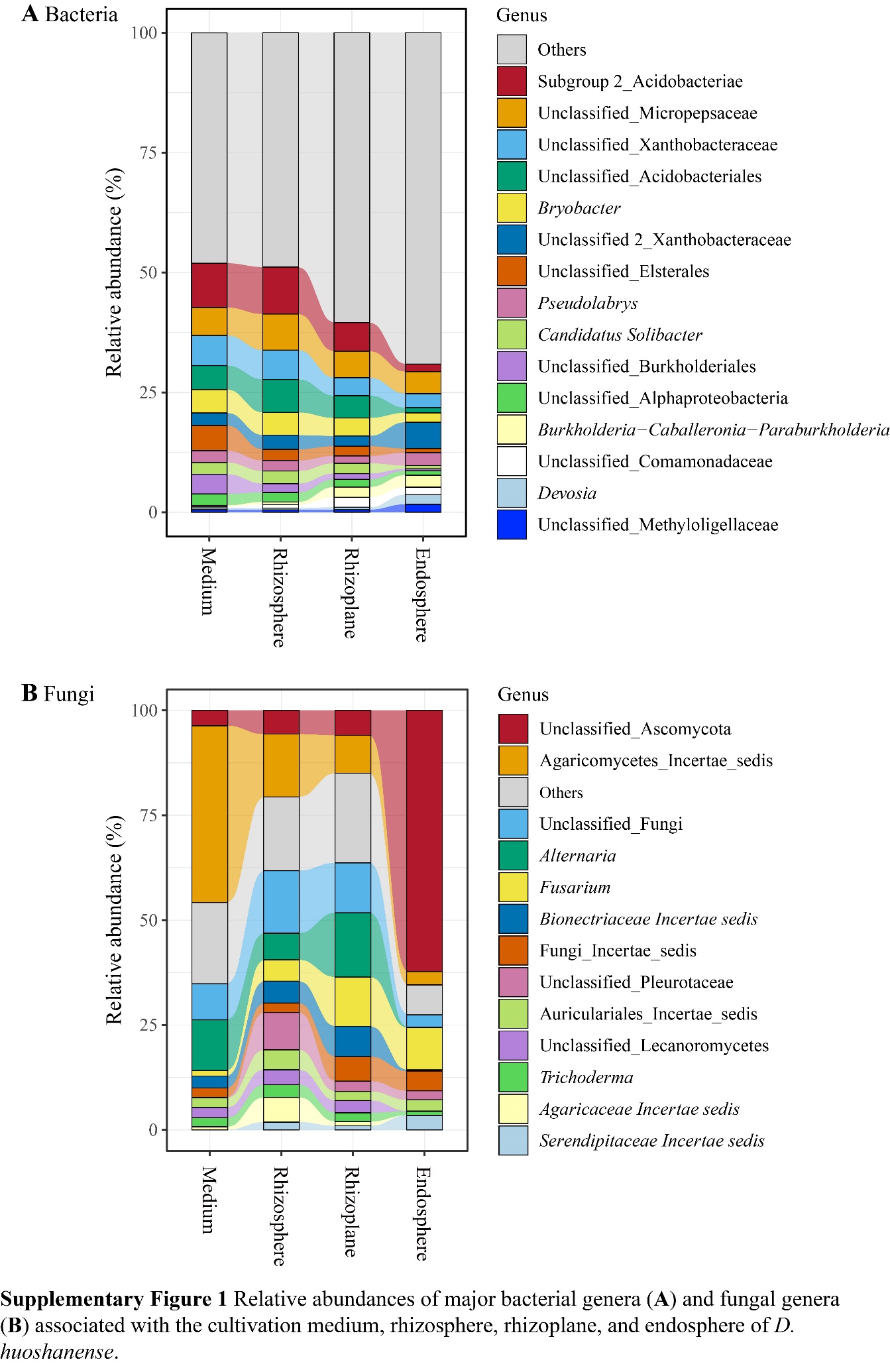


**Supplementary Figure 2** Relative abundances of major bacterial genera (**A**) and fungal genera (**B**) associated with the cultivation medium, rhizosphere, rhizoplane, and endosphere of *D. huoshanense*.

**Data availability statement**

The raw sequence data reported in this study have been deposited in the Genome Sequence Archive at the BIG Data Center (<http://bigd.big.ac.cn/gsa>) under the accession number CRA017080.
